# Supplementary figures and images for: Gene expression signatures predict response to therapy with growth hormone
Source: Pharmacogenomics J. 2021 May 27;21(5):594–607. doi: 10.1038/s41397-021-00237-5 (PMC8455334; doi:10.1038/s41397-021-00237-5)

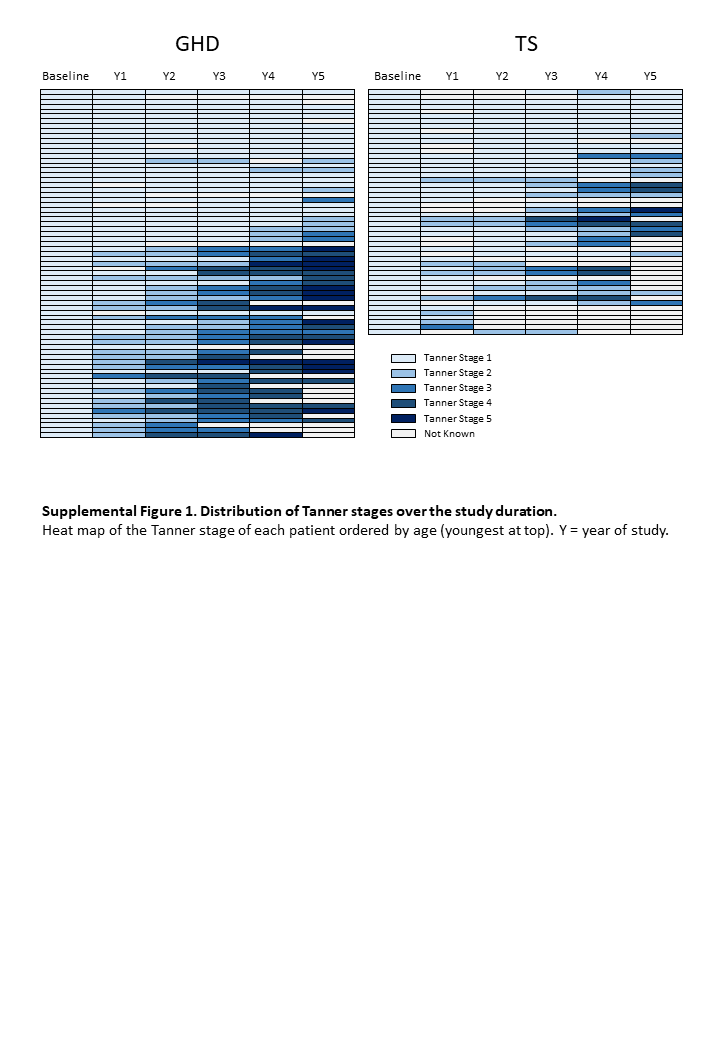

Supplement: Supplementary file 1 — Supplemental Figure 1 [file 41397_2021_237_MOESM1_ESM.tif]
